# Neck Pain in Dental Education: A Cross-Sectional Analysis of Neck Strength Differences

**DOI:** 10.3390/muscles4030040

**Published:** 2025-09-17

**Authors:** Manuel B. Almeida, Marion Moreira, Paula Moleirinho-Alves, Raúl Oliveira

**Affiliations:** 1 Neuromuscular Research Lab, Interdisciplinary Centre for the Study of Human Performance (CIPER), Faculty of Human Kinetics, University of Lisbon, 1499-002 Oeiras, Portugal; 2 Egas Moniz Center for Interdisciplinary Research (CiiEM), Egas Moniz School of Health & Science, 2829-511 Almada, Portugal; 3 Department of Physiotherapy, Egas Moniz School of Health & Science, Campus Universitario, Quinta da Granja, 2829-511 Almada, Portugal

**Keywords:** cervical pain, neck pain, dentistry students, occupational health, neck strength

## Abstract

This study aimed to investigate if there are differences in neck strength between dental students in their fourth and fifth years, with and without neck pain. Neck muscle strength was assessed in flexion, extension, and lateral flexion to both sides using a handheld dynamometer while participants performed maximum voluntary isometric contractions with a make test procedure. Differences between groups were evaluated through a one-way analysis of variance followed by Tukey’s post hoc comparison. Forty-three students (twenty-one fourth-year and twenty-two fifth-year students) participated in the study. Fifth-year students with neck pain (P5) (*n* = 14) showed less strength (*p* = 0.018) compared to the no-pain group (NP) (*n* = 15) in neck flexion and lateral flexion for both sides (*p* < 0.001). The fourth-year symptomatic students (P4) (*n* = 14) showed reduced right lateral flexion strength compared to the NP group (*p* = 0.035). No significant differences were observed in neck extension across all groups (*p* = 0.085). Our research indicates a progressive decline in neck muscle strength in flexion and lateral flexion among students with neck pain over their training years. Our findings suggest that this may be linked to the increasing mechanical demands of clinical practice during training, potentially affecting muscle balance and cervical function. This pain could be associated with changes in motor behavior and reduced cervical muscle strength.

## 1. Introduction

Musculoskeletal disorders linked to occupational activities (WMSDs) impact a large proportion of dental professionals [1], with a lifetime prevalence of 95.8% [2]. This issue has been reported as early as during their training years, with a prevalence rate varying between 44% and 93% [3,4,5,6]. These disorders are exacerbated by the demanding physical conditions of dental work [2,7], which often involve static, asymmetrical postures and repetitive movements [3,4,5]. In dental students, this physical strain predominantly affects the neck, with a reported 12-month occurrence ranging from 44 to 69% [6,8,9]. Although pain is a multifactorial phenomenon, where each unique experience, characterized by unique features, can elicit specific motor responses and alterations [10,11], cervical pain has been found to affect muscle strength [12,13,14,15,16,17,18,19,20] and motor unit activity [12,13,14,17,21,22,23] and such alterations may play a role in both the onset and worsening of persistent neck pain [14,24]. Work-related physical demands have been linked to a dose–response effect, with higher physical demands increasing the odds of developing neck, shoulder, and low back WMSDs [25]. While total clinical training hours and the complexity of tasks performed by final-year undergraduate students can be quantified, their impact on outcomes remains largely unknown. When complex tasks involve disadvantageous muscle lengths and suboptimal joint angles, muscular strength may decrease substantially, with reductions ranging from roughly 40% to 56% [26]. This compromised strength capacity not only increases the risk of fatigue but also contributes to muscle imbalances and pain, particularly during extended periods of work.

Considering the cervical segment, there are several strength assessment methods available, with different limitations [27]. Nonetheless, a systematic review [28] demonstrated that handheld dynamometers (HHDs) offer a valid and reliable approach for measuring muscle strength, especially in healthy individuals. Recent research [29,30,31] has demonstrated strong correlations between HHD measurements and isometric strength, particularly for proximal muscle groups, making it a reliable, easy-to-administer tool for accurately assessing maximal isometric cervical muscle strength in both healthy individuals [31] and patients with neck pain [32].

With our study, we aimed to investigate if there were differences in neck strength between dental students in their fourth and fifth years, with and without neck pain in the previous week. We hypothesize that students with neck pain will exhibit lower overall cervical strength compared to those without neck pain, and that these differences will be more pronounced in fifth-year than in fourth-year students.

## 2. Results

A total of 123 students were invited to participate in the study. Of these, 43 declined to be screened for eligibility. Among the 80 students who underwent screening, 19 were excluded due to a history of musculoskeletal disorders, and 11 were excluded for being over 30 years of age. Of the 50 students who met the inclusion criteria, 7 declined to take part. Consequently, 43 participants consented and completed the data collection. Of these, 30 (69.8%) were women and 13 (30.2%) were men, with a mean age of 23.35 ± 2.36 years and a mean Body Mass Index (BMI) of 21.17 ± 2.61 kg/m^2^, with 21 enrolled in the fourth year and 22 in the fifth year. A detailed overview of their sociodemographic characteristics is presented in Table 1.

Based on the responses to the NMQ, participants were categorized into three groups. The no-pain group (NP) (*n* = 15) comprised students from the fourth and fifth years without pain, while the Pain—4th year (P4) (*n* = 14) and Pain—5th year (P5) (*n* = 14) groups were composed of fourth- (*n =* 7) and fifth-year (*n* = 8) students with neck pain, respectively.

**Figure 1:**
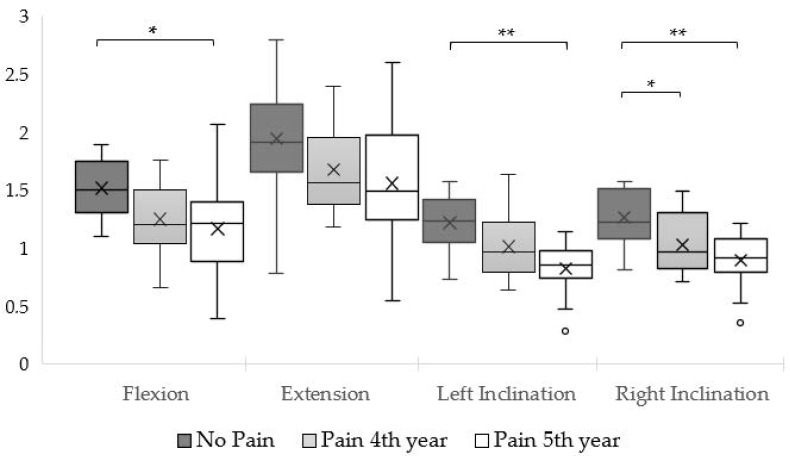
Body-weight-normalized neck strength. X = mean; * = *p*-value < 0.05; ** = *p*-value < 0.001.

For neck flexion [F_(2, 40)_ = 4.52; *p* = 0.017; *η*^2^*p* = 0.184], we found the P5 group had less strength than the no-pain group (*p* = 0.018). The same pattern was observed in left lateral flexion [F_(2, 40)_ = 8.89; *p* < 0.001; *η*^2^*p* = 0.308], with a difference between the NP and P5 groups (*p* < 0.001), and right lateral flexion [F_(2, 40)_ = 8.57; *p* < 0.001; *η*^2^*p* = 0.300], but for this movement, both the P5 (*p* < 0.001) and P4 (*p* = 0.035) groups had less strength than the NP group. Neck extension [F_(2, 40)_ = 2.62; *p* = 0.085; *η*^2^*p* = 0.116] followed a similar tendency but without statistical significance. Figure 1 illustrates the comparative neck strength across groups for different movements.

Table 2 compares the neck strength ratios of agonists to antagonists for the three study groups to established normative values for healthy 18–35-year-olds [33].

## 3. Discussion

Dental students face a high prevalence of WMSDs due to the physical demands of their education and limited experience [3,4,5,6,34,35]. The neck is the most commonly affected anatomical region, making it a critical issue for this student group and stakeholders [2,3,4,5,7,35,36]. Cervical pain has been shown to influence both muscle strength [12,13,14,15,16,17,18,19,20] and motor unit behavior [12,13,14,17,21,22,23], and such alterations are linked to the onset and progression of persistent musculoskeletal neck disorders [14,24]. Cervical musculature plays a key role in supporting the head’s weight and performing isometric contractions with sufficient duration and force to maintain stability in this region. Consequently, reduced strength and endurance of these muscles can increase mechanical load on cervical joints and soft tissues, which may contribute to the persistence of neck pain [12,13,14,15,16,17,18,19,37]. Activities such as dental practice could exacerbate those symptoms, given their associated physical demands. The persistence of pain in patients without structural damage remains poorly understood [38,39,40,41]. Comprehensive and multidimensional research is essential to fill this knowledge gap [6,35,40,41,42]. With our study, we addressed this gap by investigating the differences in neck strength between dental students in their fourth and fifth years, with and without neck pain.

We found a significant decrease in neck strength in fourth-year students compared to the no-pain group for right lateral flexion. For fifth-year students, neck strength was significantly lower in flexion, left lateral flexion, and right lateral flexion compared to the no-pain group, with large to very large effects for these movements (*η*^2^*p* = 0.184–0.308). These effect sizes indicate that group differences explained between 18% and 31% of the variance in strength, highlighting the clinical relevance of these findings beyond statistical significance [43]. In contrast, neck extension showed a similar tendency but did not reach statistical significance; nevertheless, the effect size (*η*^2^*p* = 0.116) suggests a potentially meaningful trend that may not have been detected due to limited power. These findings are consistent with prior research demonstrating a decline in neck strength among individuals experiencing neck pain [44,45]. We expected to observe strength differences in both neck pain groups compared to the no-pain group. However, significant strength reduction was primarily observed in fifth-year students with neck pain, which might reflect a cumulative effect of prolonged clinical training, higher exposure to stressors, or more persistent pain. A possible explanation for these differences could be attributed to the increased hours of clinical practice these students undergo. Students begin their fourth year with around 15 h of clinical practice per week. In the fifth year, their training typically intensifies, with practice hours often doubling and involving more complex clinical procedures. However, the total number of hours cannot be precisely quantified or standardized, as students may also engage in parallel projects or extend their practice during intervals between formal training stages to maintain clinical operations. Our findings suggest that the increased exposure to the clinical setting during the later stages of training may be associated with the lower neck strength observed in fifth-year students compared to fourth-year students. While the cross-sectional design does not allow us to confirm a progressive decline over time, the pattern is consistent with the possibility that prolonged clinical demands contribute to reduced neck strength. Since sustained physical strain on the neck in dental practices may lead to neuromuscular adaptations [20,23,46], this could underpin the observed deficit between fourth- and fifth-year students. Prior research has suggested that abnormal cervical motor behavior can contribute to the persistence of chronic musculoskeletal neck pain, with consistent mechanical irritation of cervical structures and muscle fatigue being potential contributors [47,48,49,50,51]. Our findings reinforce the increasing body of research associating chronic musculoskeletal neck pain with altered neuromuscular control [13,20,23,46,50,51,52]. The observed neck strength differences might represent a consequence of those changes, as they could influence the muscular performance of the neck region. The disruption of the delicate balance between agonist and antagonist muscles can lead to cervical insufficiency [45,51,53]. The agonist/antagonist muscle ratio is a critical parameter for maintaining cervical stability. An imbalance in this ratio may indicate neuromuscular adaptations, which can influence the biomechanical load distribution across cervical structures. Such imbalances may predispose individuals to instability, muscle fatigue, and chronic pain symptoms [45,51,53]. In our study, students in the no-pain group exhibited an increased agonist/antagonist ratio for neck flexors compared to normative values for their age and sex [33]. The male (0.73) and female (0.81) students without pain showed an increased influence of neck flexors when compared to the normative values (0.64) [33]. This could represent the neuromuscular adaptation needed for cervical stabilization associated with their clinical practice. One potential explanation for this phenomenon is the recurrent engagement of neck extensors in dental practices. This continuous activity implies sustained contractions of the neck extensors and flexors to maintain cervical stability. Current evidence indicates that neck flexors possess approximately 64% of the strength of neck extensors for this age group [33]. However, in dental students, within the NP group used as control, we saw it ranging between 73% and 81% in males and females, respectively. Therefore, this consistent demand seems to enhance the strength and endurance of both muscular groups, with a greater effect in the neck flexors as the muscle group with lower strength, resulting in the increased agonist/antagonist ratio observed in the no-pain group. However, when we compared the NP group with the P4 (0.66) and P5 (0.68) groups, we observed a decrease in the flexion/extension ratio. The observed changes in strength ratios may be indicative of neuromuscular adaptations previously described in the literature [23,51,53]. However, further studies using electromyography and other methods are needed to confirm these hypotheses. This observation could reflect a potential relation between dental activities and the functional role of neck flexors. An increased co-activation of the antagonists was previously reported in patients with neck pain [23,51], and the enhanced co-contraction of antagonists reduces force output, but it also impairs, by reciprocal inhibition, the ability to fully activate the agonists [53]. The observed adaptations could reflect a potential motor control adjustment, as the neck pain associated with the mechanical demands of the students’ clinical work may influence greater reliance on the neck extensors. This could reduce the activation capacity of the neck flexors, leading to a lower flexor–extensor ratio compared to the NP group. The reduced flexor–extensor ratio observed in the pain groups suggests a shift in cervical muscle dynamics, potentially leading to compromised cervical stability that could potentially exacerbate pain symptoms in students who engage in clinical practice. However, despite these previously described proposed explanations [51,53,54], it is important to keep in mind that training-related increases in muscular strength may or may not result in greater co-activation, and surface electromyography activity should also be monitored [54,55] to confirm these hypotheses.

In our study, strength ratios between left and right neck lateral flexion in dental students without pain were consistent with reference values [33], despite overall higher strength measurements. This could indicate that clinical practice does not have a major influence on this agonist/antagonist ratio. However, a different pattern was noted in the fifth-year students experiencing neck pain, where an imbalance favoring the right-side muscles was observed. While the underlying reasons for this asymmetry remain unclear, the findings align with previous evidence showing side-specific differences in cervical muscle function among individuals with neck pain [56]. In this population, this may be associated with the typical posture adopted by students during patient interactions, which often involves a combination of cervical flexion with left lateral flexion and rotation. Current evidence shows that a maintained cervical rotation involves an augmented muscle activity of the contralateral flexors [56], and the pain is often related to impairments in neck strength and altered activation patterns [54]. Such a sustained, non-neutral position could potentially lead to the observed muscular imbalance, particularly in students who have accumulated a greater number of clinical training hours as observed. The maintained ratio in the P4 group and the observed difference in the P5 group could indicate a possible long-term impact of clinical practice on these students’ neck strength. This disparity may be reflective of the underlying mechanisms previously described [51,53], where increased co-activation of antagonist muscles was reported in patients with neck pain. This suggests that prolonged engagement in specific postural practices during dental procedures might contribute to the development of muscular imbalances over time as a result of these compensatory strategies or protective responses.

Therefore, our findings align with previous research suggesting that the mechanical overload associated with clinical practice may be associated with the decline in neck strength observed among dental students already experiencing pain. During certain periods of the year, fifth-year students experience a significant increase in clinical training hours compared to their fourth-year counterparts. This increased workload may enhance motor behavior adaptations in students experiencing pain and contribute to the development of non-specific neck pain, which has been associated with muscle inhibition [17,21,22,57], altered movement patterns [18], and cervical muscle weakness [17,18,19,20,45].

Our study contributes to the ongoing debate regarding the association between pain side and strength decline in neck muscles. Existing evidence is mixed, with some studies indicating strength changes exclusively on the symptomatic side [15,58], while others report bilateral effects [16]. Although specific information about the side of pain was not collected, our findings reveal a consistent pattern in both left and right lateral flexion, supporting the notion of bilateral strength decrease due to pain. Nonetheless, we observed reduced neck strength in both fourth- and fifth-year students during right lateral flexion movements, which could be attributed to the asymmetrical position during clinical training. In our university setting, students approach the patient from the patient’s right side, often maintaining a position that combines neck flexion with left lateral flexion and rotation. This posture leaves the neck extensors and right lateral flexors exposed to repeated and sustained contractions to stabilize the neck against gravity. This might explain the absence of significant differences in neck extension across all groups, and the tendency to an impairment of their antagonists represented by the agonist/antagonist ratios changes observed, especially in the fifth-year students with neck pain.

Despite our efforts to address previously identified shortcomings, our study faced several limitations. We were unable to explore the influence of the multifactorial dimension of pain over the neck strength. Additionally, the no-pain group containing students from their fourth and fifth years may restrict the depth of our analysis and the generalizability of our findings since it may have masked potential differences in neck strength between these two academic years, limiting our ability to detect more subtle year-specific effects. In addition, the relatively small sample size and sex imbalance, with approximately 70% of participants being women, while representing the normal sex distribution in this populations, may limit the generalizability of our findings and reduce statistical power to detect more subtle effects. The cross-sectional nature of our study design also limits our ability to identify predictors and establish causal links between neck pain and muscle strength. To address these gaps, future research should include longitudinal designs with larger samples to understand the functional adaptations of strength and activation patterns during clinical training years as previously recommended [55]. These methods can offer a more comprehensive understanding of the neuromuscular adaptations experienced by dental students and their potential impact throughout their academic training.

## 4. Materials and Methods

### 4.1. Study Design

This cross-sectional study received ethical approval from the Egas Moniz School of Health & Science Ethics Committee (CEEM-1122, September 2022) and adhered to ethical principles outlined in the Declaration of Helsinki (2008). Data was anonymized and will be deleted in April 2028. The study was designed and reported according to STROBE guidelines [59].

### 4.2. Participants

In order to reduce unnecessary displacements and to minimize potential disruptions to the participants’ academic and clinical schedules, a convenience sampling strategy was adopted. Participants were recruited on a voluntary basis from the population of fourth- and fifth-year dental students enrolled in the master’s degree program at Egas Moniz School of Health & Science, Portugal. This approach ensured accessibility to the study cohort while maintaining feasibility within the educational setting. Based on responses to the Nordic Musculoskeletal Questionnaire (NMQ), students were divided into three categories: those without neck pain (NP), fourth-year students reporting neck pain (P4), and fifth-year students reporting neck pain (P5). To ensure the rigor of the research, group allocation was concealed from both investigators and assessors. Inclusion was limited to clinically active dental students. Exclusion criteria included age over 30, pre-existing diagnosed musculoskeletal disorders, inability to provide informed consent, communication barriers, recent fractures, major neurological or musculoskeletal conditions, central or peripheral nervous system dysfunctions, and diagnosed psychiatric or substance abuse disorders.

To ensure adequate statistical power, a sample size of 42 participants, with 14 students per group, was calculated using G*Power software (version 3.1), considering an alpha level of 0.05, a power of 0.80, and a medium effect size of 0.5 [60].

### 4.3. Procedure

An online form was used to obtain informed consent and administer the NMQ [61] for identifying students with neck pain in the previous week and allow group allocation [20]. The questionnaire was administered at the beginning of the second semester, from January to March. Informed consent was obtained prior to NMQ completion and strength testing.

At baseline, all participants underwent a preliminary evaluation to collect demographic and anthropometric characteristics, including age, sex, body mass, height, and Body Mass Index (BMI). Following this initial characterization, the strength assessments were carried out using the KForce Bubble Pro dynamometer (Kinvent, Montpellier, France), a portable, battery-powered instrument specifically designed to quantify peak muscle force. The device operates on a strain gauge-based load cell mechanism, in which the application of force induces deformation of the gauge, generating an electrical signal proportional to the exerted force. This signal is then processed and displayed through the dedicated Kinvent smartphone application. The dynamometer provides real-time measurements expressed in kilogram-force (kgf) or Newtons (N), with a measurement capacity of up to x N, thereby allowing for precise and reproducible quantification of muscular output.

To ensure methodological rigor, all examiners underwent structured training in the standardized use of the KForce dynamometer under the supervision of an experienced physiotherapist who possessed prior expertise with the equipment. Before initiating formal data collection, a pilot study involving four participants was conducted. This preliminary stage served to refine testing procedures, familiarize assessors with the protocol, and confirm the reliability of the assessment setup.

**Figure 2:**
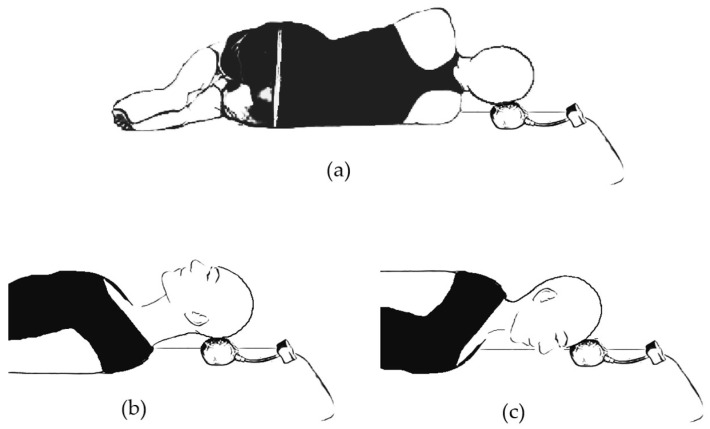
Cervical strength assessment positions: (**a**) side-lying for neck lateral flexors, (**b**) supine for neck extensors, and (**c**) prone for neck flexors [20].

The formal evaluations were conducted individually at the Egas Moniz University Clinic in a controlled environment to enhance consistency and reduce the influence of external confounding variables. Testing sessions were performed in a dedicated room maintained under standardized ambient conditions, with room temperature regulated between 21 °C and 23 °C and relative humidity controlled within the range of 40% to 60%. All assessments took place in the morning, between 9:00 and 13:00, to minimize the potential impact of circadian fluctuations in muscle performance and neuromuscular function. Participants were instructed to comply with specific pre-test requirements to standardize physiological conditions across the sample. These included abstaining from strenuous physical activity and alcohol consumption for at least 24 h prior to testing, as both factors are known to influence neuromuscular function and fatigue levels. Additionally, participants were asked to refrain from food intake for a minimum of three hours before the assessment, thereby minimizing potential metabolic variability or discomfort that could interfere with test performance. Cervical muscle strength, including flexion, extension, and bilateral lateral flexion, was assessed using a KForce Bubble Pro dynamometer during maximal voluntary isometric contractions (MVICs), in accordance with current methodological standards [20,62]. Prior to testing, participants received standardized instructions and familiarization training to ensure consistent execution of the testing procedures. Strength evaluation employed a “make test” protocol, in which participants performed three consecutive MVIC trials lasting 5 seconds each, separated by 30 s rest intervals. The highest recorded force value across the three trials was used for analysis [20]. The “make test” was selected over alternative protocols as it has been shown to reduce the influence of examiner strength, improve inter-rater reliability, and minimize the risk of injury during testing, particularly in the cervical region [20,62]. The make test was carried out by the examiner holding the dynamometer stationary while the participant exerted a maximal force against it. Cervical flexion strength was measured with participants in a prone position, extension in supine with the arms aligned alongside the trunk, and lateral flexion in a side-lying position with the hips and knees flexed to 45° and the arms crossed over the chest. To limit variability associated with posture and stabilization, participants were instructed to progressively increase contraction during the initial second, followed by a four-second phase of maximal effort. During this phase, standardized verbal encouragement was provided (“push as hard as you can, as hard as you can”). The testing order for flexion, extension, and lateral flexion was randomized across participants to mitigate sequence or fatigue effects. The assessment positions are illustrated in Figure 2.

To mitigate the influence of body size on muscle strength measurements and ensure reliable comparisons, we employed the allometric method [63] to normalize strength data rather than reporting joint torque, a widely accepted approach in musculoskeletal research to facilitate comparability across participants [63]. We used the equation S*_n_* = S/m^b^, where S*_n_* (normalized strength) was calculated by dividing the recorded muscle force (S) by the participant’s body mass (m). The allometric parameter (b) should be b = 0.67 for muscle force recorded by a dynamometer to assess the body-size-independent indices of muscle strength [63].

### 4.4. Statistical Analysis

All statistical analyses were conducted using Jamovi software (version 2.4.8). Group comparisons for sex distribution were examined using the non-parametric Chi-square test. Continuous variables, including age, height, body mass, and Body Mass Index, were compared across groups using a one-way analysis of variance (ANOVA). Prior to performing ANOVA, the assumptions of normality and homogeneity of variances were assessed using the Shapiro–Wilk and Levene tests, respectively. When these assumptions were satisfied, one-way ANOVA followed by Tukey’s post hoc procedure was applied to identify pairwise differences between groups. Statistical significance was set at a two-tailed alpha level of 0.05.

## 5. Conclusions

Dentistry students with neck pain have a gradual decrease in muscle strength in neck flexion and lateral flexion for both sides during their training years. While the accumulation of training hours and weekly hours of clinical practice may contribute to these changes, our study’s cross-sectional design does not allow for causal relationships to be established. Our findings highlight the potential influence of increased mechanical demands of clinical practice on neck muscle function and agonist/antagonist muscle ratios in students with neck pain. Based on these findings, it may be worthwhile for future research to explore the effectiveness of integrating ergonomics training and targeted exercises into dentistry curriculum as a potential strategy to mitigate neck strength declines and promote long-term musculoskeletal health among students.

## Figures and Tables

**Table 1:** Overview of participants’ demographic features.

	No Pain (*n =* 15)		Pain—4th Year (*n* = 14)		Pain—5th Year (*n* = 14)		
	Mean (+SD)	[Range]	Mean (+SD)	[Range]	Mean (+SD)	[Range]	*p*
Age (years)	22.9 (±1.49)	[20–26]	22.8 (±2.26)	[20–28]	24.6 (±3.48)	[22–30]	0.088 ^a^
Height (cm)	169.3 (±9.08)	[156–183]	168.1 (±11.19)	[152–196]	170.9 (±9.4)	[153–190]	0.751 ^a^
Body Mass (kg)	63.7 (±3.44)	[46–88]	61.7 (±13.1)	[46–96]	58.2 (±10.12)	[43–83]	0.338 ^a^
BMI (kg/m^2^)	22.0 (±3.01)	[18.1–26.9]	21.6 (±2.33)	[17.1–25.0]	19.8 (±1.96)	[17.4–23.0]	0.103 ^a^
Sex % (*n*)	Male 46.7% (7) Female 53.3% (8)		Male 21.4% (3) Female 78.6% (11)		Male 21.4% (3) Female 78.6% (11)		0.229 ^b^

Note. BMI = Body Mass Index; SD = Standard Deviation; ^a^ One-way ANOVA; ^b^ Chi-square test.

**Table 2:** Neck strength ratios.

Ratios	Reference [33]		No Pain		P4		P5	
	**F/E**	**L/R**	**F/E**	**L/R**	**F/E**	**L/R**	**F/E**	**L/R**
Male	0.64	1.00	0.73	0.98	0.66	1.02	0.68	0.91
Female	0.64	0.98	0.81	0.99	0.78	0.97	0.78	0.92

Note. F/E = flexion/extension ratio; L/R = left [lateral flexion/right lateral flexion ratio; P4 = fourth year with neck pain; P5 = fifth year with neck pain.

## Data Availability

The dataset generated and analyzed during the current study is publicly available in the Mendeley Data repository at the following link: https://data.mendeley.com/datasets/hm7xw26kjd/1 (accessed on 2 January 2024).

## Abbreviations

The following abbreviations are used in this manuscript:

ANOVA	Analysis of Variance
BMI	Body Mass Index
CEEM	Egas Moniz School of Health & Science Ethics Committee
F/E	Flexion/Extension ratio
HHD	Hand-Held Dynamometer
L/R	Left lateral flexion/Right lateral flexion ratio
MVIC	Maximal Voluntary Isometric Contraction
NMQ	Nordic Musculoskeletal Questionnaire
NP	No Pain (group)
P4	Pain–4th year (group)
P5	Pain–5th year (group)
SD	Standard Deviation
STROBE	Strengthening the Reporting of Observational Studies in Epidemiology
WMSDs	Work-Related Musculoskeletal Disorders
